# Origin of the Nuñoa, Perú High Altitude Field Research Site and How It Shaped Our Understanding of Functional Adaptation to High‐Altitude Stressors

**DOI:** 10.1002/ajhb.70031

**Published:** 2025-04-12

**Authors:** A. Roberto Frisancho

**Affiliations:** ^1^ Department of Anthropology and Center for Human Growth and Development University of Michigan Ann Arbor Michigan USA; ^2^ National University of San Antonio Abad Cusco Peru

## Abstract

The study of physical growth and development of Indigenous children from Nuñoa, Perú, in the 1960s showed that growth in body size and skeletal maturation was slow and delayed, while growth in lung volume, measured by forced vital capacity (FVC), was accelerated. Hence, I proposed that the high functional adaptation of high‐altitude natives was influenced by developmental processes. To test this hypothesis, my co‐investigators and I conducted two sets of major physiological studies at high altitudes. The first studies were conducted in Cusco (3400 m) and Puno (3840 m), Perú. This research showed that the FVC and aerobic capacity of low‐altitude Peruvian urban natives acclimatized to high altitudes during the developmental period were similar to those of high‐altitude urban natives. In contrast, Peruvian and US participants acclimatized during adulthood did not have the same FVC and aerobic capacity as the high‐altitude urban natives. The second set of studies was carried out in the city of La Paz, Bolivia (3752 m), and included Europeans who were acclimatized to high altitudes at different ages. This research confirmed that acclimatization during the developmental period was a major component of the high functional adaptation among high‐altitude urban natives. These conclusions have been confirmed by epigenetic studies, which demonstrated that acclimatization to high altitude leads to modifications in the activity of the DNA that facilitate adaptation during the developmental period.

## 1 Introduction

This report consists of two sections. The first part provides my personal account of how the field site of Nuñoa, Perú, came to be the center for high‐altitude research as part of the Human Adaptability Program (HAP) of the International Biological Programme (IBP) in the 1960s and early 1970s. Thereafter, I re‐examine my research on human growth in Nuñoa and its influence on subsequent research in Perú and Bolivia addressing the role of developmental factors in the attainment of high functional adaptation among high‐altitude urban natives.

## 2 A Personal Account of the Origins of the Nuñoa, Perú High Altitude Research Field Site

**FIGURE 1:**
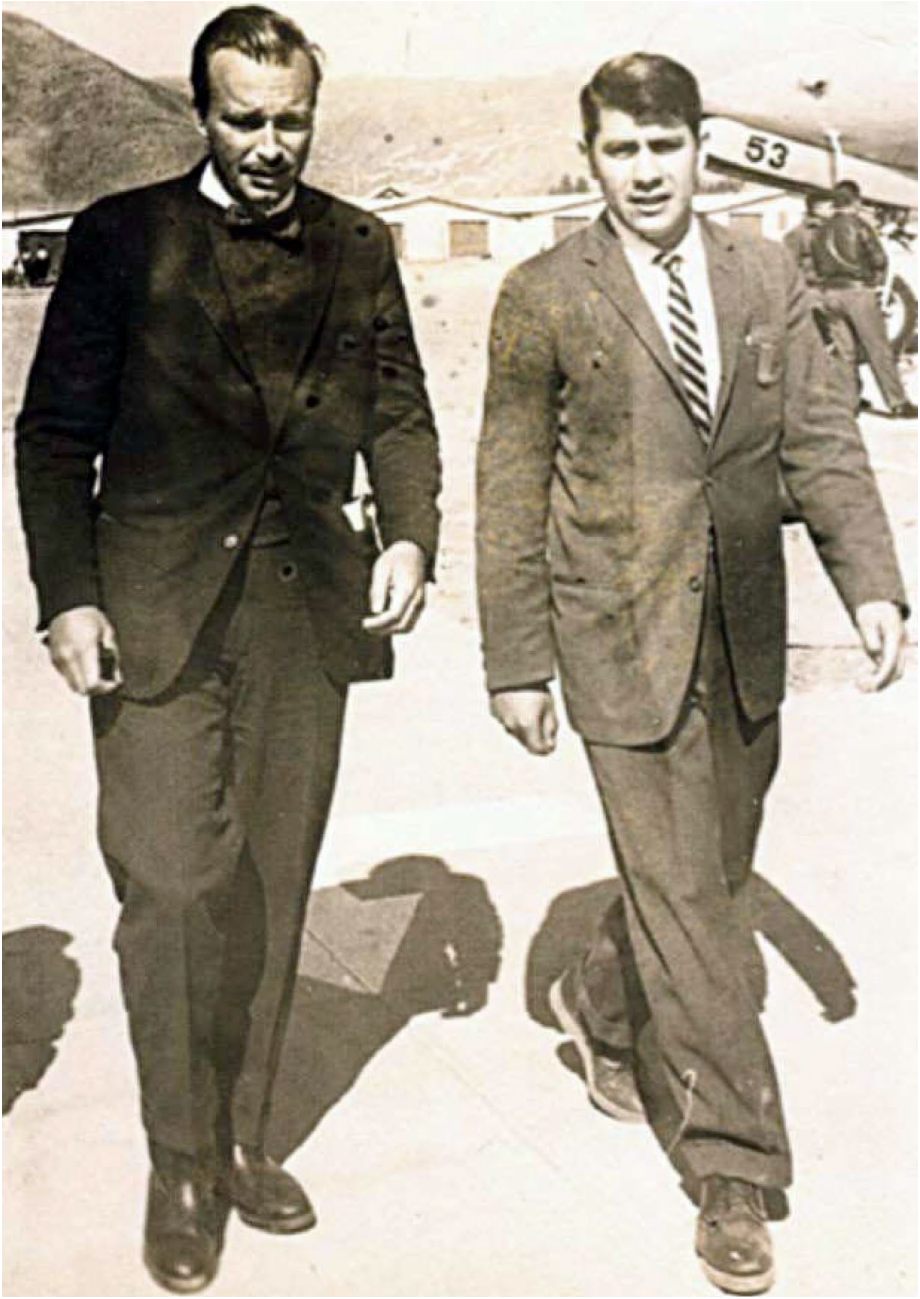
A. Roberto Frisancho (on the right) meeting Paul T. Baker at the Cusco, Perú airport in May 1961. This encounter marks the start of research on human bio‐cultural adaptation in Nuñoa, Perú.

In May of 1961, upon the request of Dr. Sergio Quevedo of the National University of San Antonio Abad (UNSA) in Cusco, Perú, I went to the Cusco airport to meet a US Fulbright Scholar named Paul T. Baker of the Pennsylvania State University (see Figure 1). Paul arrived with his wife, Thelma, and their four children (Debby. Amy, Joshua, and Felecia). At the airport, Paul asked me about my background, and I told him that I was a third‐year student in Humanities and Anthropology at UNSA. I also noted that I was fluent in Quechua and Spanish and was learning English and French while studying at the Tour Guide School of Cusco.

Baker indicated that the purpose of his trip *was to study how the Andean Quechua natives adapt to the chronic cold stress*. Thereafter, he hired me as his Research Assistant to join his team that included four of his graduate students: Richard Mazess, Michael Little, J. Michael Hanna, and Lois Eidenof. Ultimately, the research team was rounded out with one additional RA, Julio Sotomayor, a classmate of mine at the Tour Guide School of Cusco.

To carry on with this research, Baker indicated that we needed to find an Indigenous population living near Cusco. We settled on the town of Chinchero (3450 m), located about 30 km from Cusco. One of my cousins was the Director of the new elementary school in the town. He graciously gave us access to two of the school's classrooms that were not yet occupied. Richard Mazess converted the classroom into a cold‐testing lab that could maintain temperatures of 10°C and 14°C. The tests conducted in Chinchero included measurements of rectal temperature, skin temperature at the chest, hands, and feet for 2 h at temperatures of 10°C or 14°C. The study also included measurements of finger temperature while immersed in ice water. Baker (1963) presented the details of the research protocol and the initial findings.

By his second month in Perú, Baker came to realize that cold stress was not likely the major selective force that would induce genetic adaptation. Instead, he viewed high‐altitude hypoxia as the major environmental stress that needed to be studied. He then decided to fly to Lima to discuss this with Dr. Alberto Hurtado, the leading high‐altitude physiologist at the Universidad Cayetano Heredia. Upon his return to Cusco, Baker indicated that future research would address adaptation to *both* high‐altitude hypoxia and cold stress. Further, he noted that this work should be conducted at higher altitudes than Cusco, in a community further from major urban centers.

Again by coincidence, I had connections with families living in the remote *altiplano* town of Nuñoa (4240 m) in the Department of Puno. The family of my high school classmate, Victor Barreda, owned a farm in the Nuñoa District. After discussing the proposed research with the Barredas and Dr. Manuel Chavez Ballón (an archeologist conducting research in Nuñoa), Paul and Thelma Baker, Chavez Ballón, and I visited Nuñoa to speak with community leaders. Baker concluded that this was the ideal place to carry out long‐term research and purchased a piece of land from the Barredas to build the future Nuñoa Research Laboratory.

In sum, Paul Baker and his research team initially came to Cusco to determine whether the Indigenous populations of the Andes were “mesothermic” or “hyperthermic” in their responses to cold stress. Baker soon realized that his research program was too narrow and needed to be expanded to include considerations of hypoxia and other high‐altitude stressors. This shift in orientation and focus represents an important milestone in the development of the field of human population biology. The establishment of the field site in Nuñoa laid the foundations for the first major biocultural research on human adaptability to high‐altitude environments as part of the HAP‐IBP (see Baker and Little 1976).

## 3 The Study of Human Growth in Nuñoa

After graduating in March 1963 with a BA in Humanities from UNSA, I went to the Pennsylvania State University to start my graduate studies in Biological Anthropology with support from the Fulbright Foundation. For my master's and PhD theses, I conducted research in Nuñoa from 1966 to 1967. The work involved: (a) anthropometric assessment of physical growth and development, (b) assessment of skeletal maturation using hand‐wrist radiographs, and (c) assessment of growth in lung volume by measuring forced vital capacity (FVC).

### 3.1 Slow Growth in Stature

**FIGURE 2:**
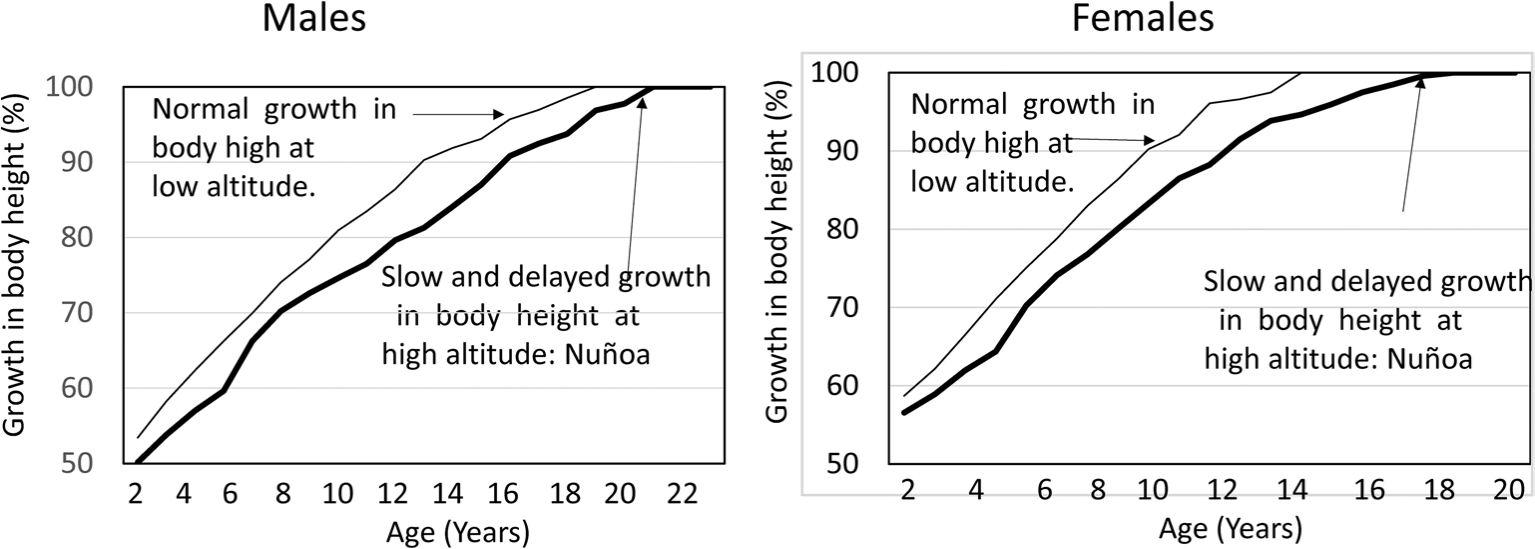
Slow growth of children from Nuñoa expressed as a percent of adult body height. Growth in body height of both males and females is slow and delayed compared to low‐altitude references. *Source:* Adapted from: Frisancho and Baker (1970).

The study of physical growth in Nuñoa was based on a total sample of 1202 Quechua participants between the ages of 2 and 35 years. The data also included a mixed longitudinal sample of 300 individuals between 1 and 22 years of age. Figure 2 shows the growth in stature among Nuñoan boys and girls compared to US reference data (adapted from Frisancho and Baker 1970). The graphs highlight the delayed growth of the high‐altitude children compared to their low‐altitude counterparts. Nuñoan males attained full adult stature at about the age of 20 and 22 years, whereas Nuñoan females achieved their adult height at the age of 18 years. Skeletal maturation and the timing of the adolescent growth spurt were similarly delayed among the children of Nuñoa (Frisancho and Baker 1970). Similar growth delays associated with high altitude have been documented among urban children from La Paz, Bolivia (Greksa et al. 1984).

Subsequent research in Nuñoa has documented that much of the growth stunting and delays in physical growth were attributable more to nutritional stress than hypoxia (see Leonard 1989; Leonard et al. 1990; Leatherman et al. 1995; Pawson and Huicho 2010). These findings have been largely confirmed by recent work in Nuñoa documenting a marked secular trend in statural growth between 1999 and 2015 associated with improvements in economic well‐being and nutritional health (Hoke and Leatherman 2019). Parallel findings of the influence of socioeconomic status on statural growth have been reported among children of the communities of Tintaya (4100 m) and Marquiri (4100 m) in the southern Peruvian highlands by Pawson et al. (2001). Together, these results suggest that the delayed physical growth and maturation of Nuñoa children initially documented in the 1960s was largely due to nutritional factors.

### 3.2 | Accelerated Growth in Lung Volume

**FIGURE 3:**
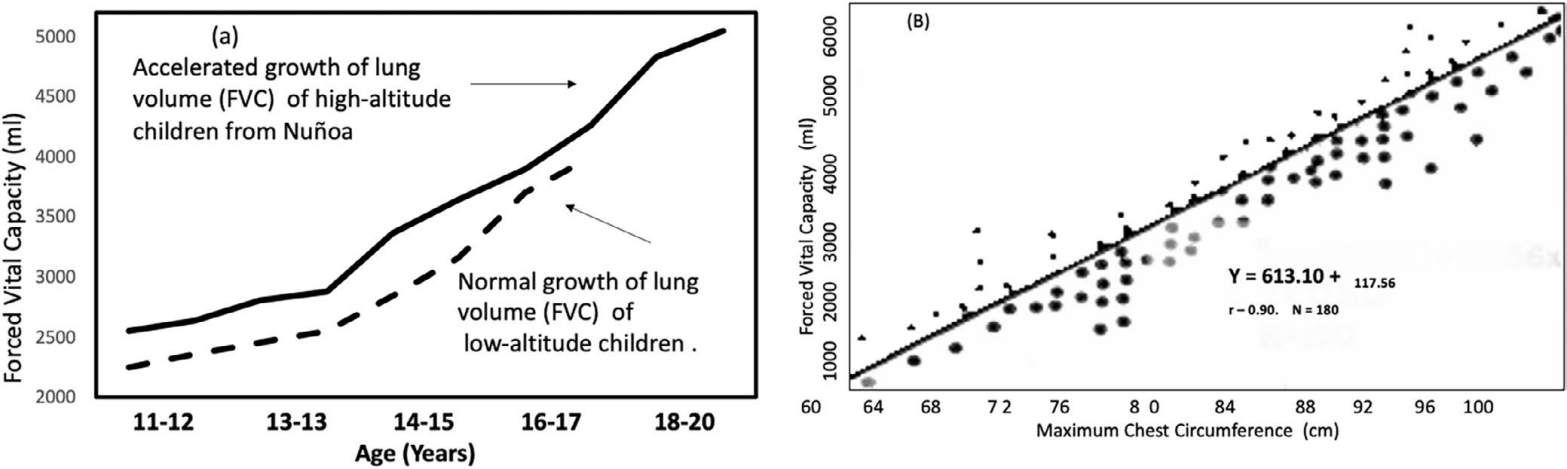
Growth in lung volume and its relationship to chest circumference in Nuñoa. The development of the size of the lungs is accelerated, which in turn is related to an enhanced chest size. *Source:* Adapted from: Frisancho (1969).

The study of the development of lung volume was based on the evaluation of forced vital capacity (Frisancho 1969), The forced vital capacity (FVC) represents maximum volume of air that a participant can exhale forcefully after taking a full Inspiration. The study included a cross‐sectional sample of 150 Quechua boys ranging in age from 11 to 20 years. A as shown in Figure 3a, despite the slow growth in stature, the FVC of the children from Nuñoa grew at a faster rate than the US reference (Bjuree 1963). The lung volumes of Nuñoa children were also greater than the FVC derived from predictive equations (Hankinson et al. 1999). Furthermore, the growth in chest dimensions of Nuñoan children was also accelerated compared to the chest size of low‐altitude children from Perú (Pretto and Calderon 1947). As shown in Figure 3b, measurements of chest circumference are positively associated with FVC, suggesting there is an interdependence between respiratory function and thorax morphology in adaptive responses to high‐altitude hypoxia (Callison et al. 2022).

Llanur et al. (2013) evaluated the growth of lung volume through measurements of functional residual capacity (FRC), which measures the volume of air remaining in the lungs after a normal, passive exhalation. This study a sample of infants and toddlers born and raised at 3440 and 44 m in Tucuman, Argentina. These authors found that lung volume at high altitude was significantly greater than at low altitude, indicating that the accelerated growth in lung volume at altitude begins very early in life (Llanur et al. 2013).

In sum, growth at high altitude is characterized by a biological trade‐off that results in slow growth in body size and a compensatory, accelerated growth in lung volume.

## 4 Testing the Developmental Adaptation Hypothesis

Because there is an association between FVC (Frisancho 1969) and lung volume (Hankinson et al. 1999), an accelerated growth of FVC would be conducive to an enlarged lung volume. Early high‐altitude researchers posited that an enlarged lung volume was an important component of the high functional adaptation among Indigenous Andean populations (Velasquez and Florentini 1966; Hurtado 1964; Kollias et al. 1968; Baker 1969). Hence, I postulated that the accelerated lung growth of Nuñoan children represented a developmental response that contributed to the high functional adaptation of high‐altitude natives (see Frisancho 1970). To test this hypothesis, my co‐investigators and I conducted extensive studies in Perú and Bolivia examining the developmental components of lung volume and aerobic capacity at high altitude. The results of this work are summarized below.

### 4.1 Studies of Functional Adaptation to High Altitude in Perú: Puno and Cusco

#### 4.1.1 Evaluation of Lung Volume

Measurements of FVC were taken at two locations, in the cities of Puno (3840 m) and Cusco (3400 m). The study in Puno included 53 subjects comprised of the following groups: (a) 40 Peruvian high‐altitude natives and (b) 13 Peruvian low‐altitude natives acclimatized to high altitude as adults. The high‐altitude subjects were born and raised above an altitude of 3500 to 3700 m and lived all their lives at high altitudes. They were of highland Quechua Indian descent, and their parents were highland rural natives. The low‐altitude participants were serving on the Navy base in the city of Puno and had lived at high altitudes from 9 months to 2.3 years. They were of highland Amerindian descent, and their parents were from the lowlands.

The study in Cusco included 51 participants recruited from students attending the University of Cusco and of mixed Spanish–Quechua ancestry. The composition of this sample included: (a) 20 high‐altitude urban natives who were born and raised above an altitude of 3000 m and lived all their lives at high altitudes, (b) 21 Peruvian low‐altitude natives acclimatized to high altitude in the developmental period of childhood and adolescence (i.e., between the ages of 2 and 16 years), and (c) 10 U.S. (White) low‐altitude natives acclimatized to high altitude as adults.

**FIGURE 4:**
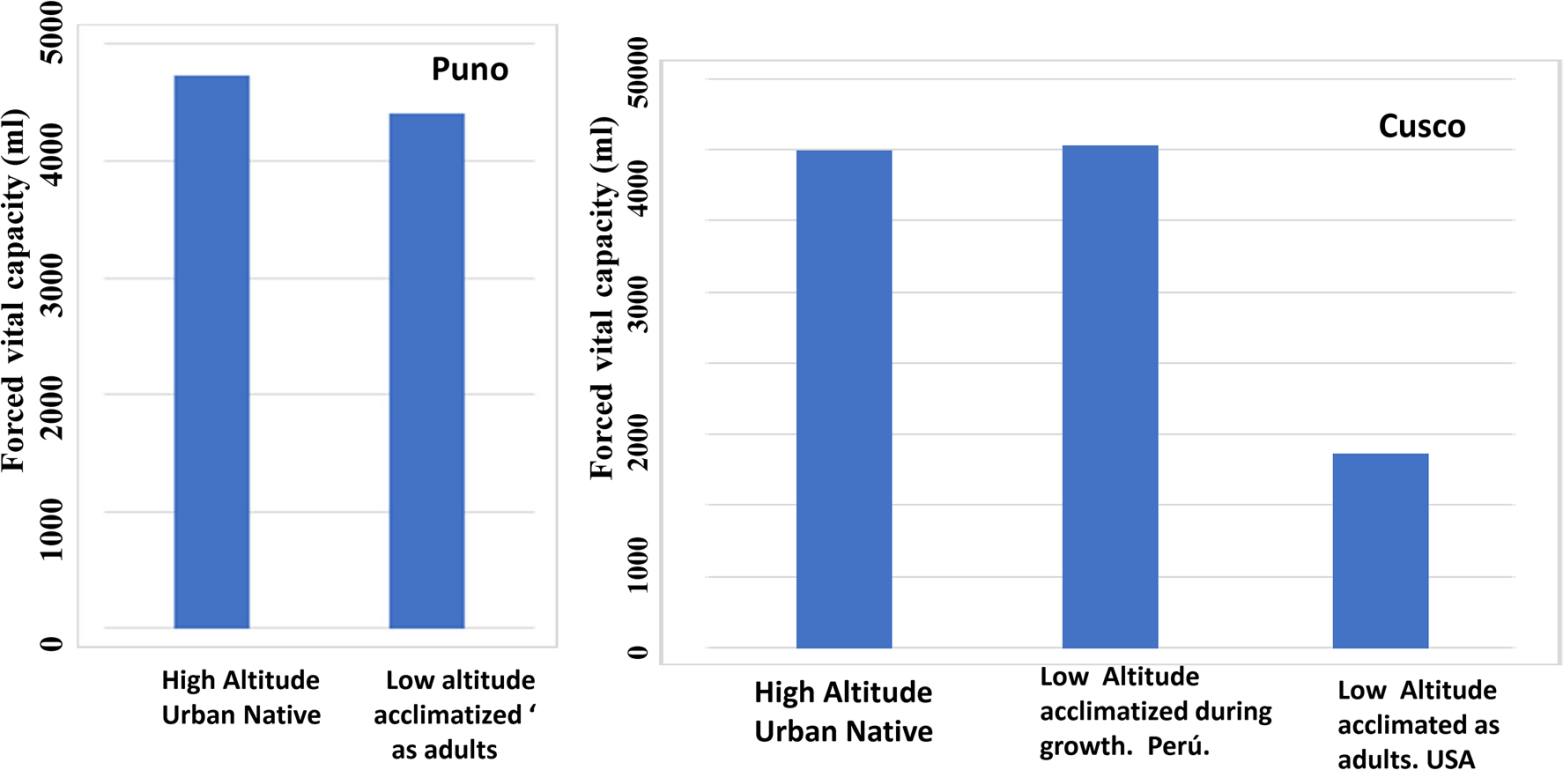
Role of developmental adaptation on the attainment of an enhanced lung volume at high altitude. Low‐altitude natives acclimatized during growth had a similar lung volume as the high‐altitude natives. In contrast, the Peruvian and US low‐altitude natives acclimatized as adults attained significantly lower FVC than the high‐altitude natives. *Source:* Adapted from: Frisancho, Velasquez et al. (1973); Frisancho (1975).

The results from the two study sites are summarized in Figure 4. In the Puno sample, the high‐altitude rural natives had a greater FVC than their low‐altitude adult acclimatized counterparts. In contrast, among the Cusco sample, the low‐altitude natives acclimatized to high altitude during the development period attained an FVC that was equal to that of the high‐altitude urban natives. In contrast, the U.S. subjects who were acclimatized to high altitude during adulthood attained significantly lower FVCs than those of the high‐altitude urban natives.

#### 4.1.2 Evaluation of Aerobic Capacity

Research on aerobic capacity was conducted among 63 young male students from the National University of Cusco. The tests were performed twice and included measurements of oxygen consumption (VO2_max_) and pulmonary ventilation (VE_max_) under maximal exercise on a bicycle ergometer. The tests were carried out at the physiological laboratory of the Regional Hospital of Cusco, situated at 3400 m. The participants included: (a) 23 Peruvian low‐altitude natives who, during the developmental period, were acclimatized to chronic high altitude, (b) 20 high‐altitude urban natives, (c) 10 Peruvian low‐altitude natives that migrated to high altitude as adults, and (d) 10 US low‐altitude natives that came to high altitude as adults.

**FIGURE 5:**
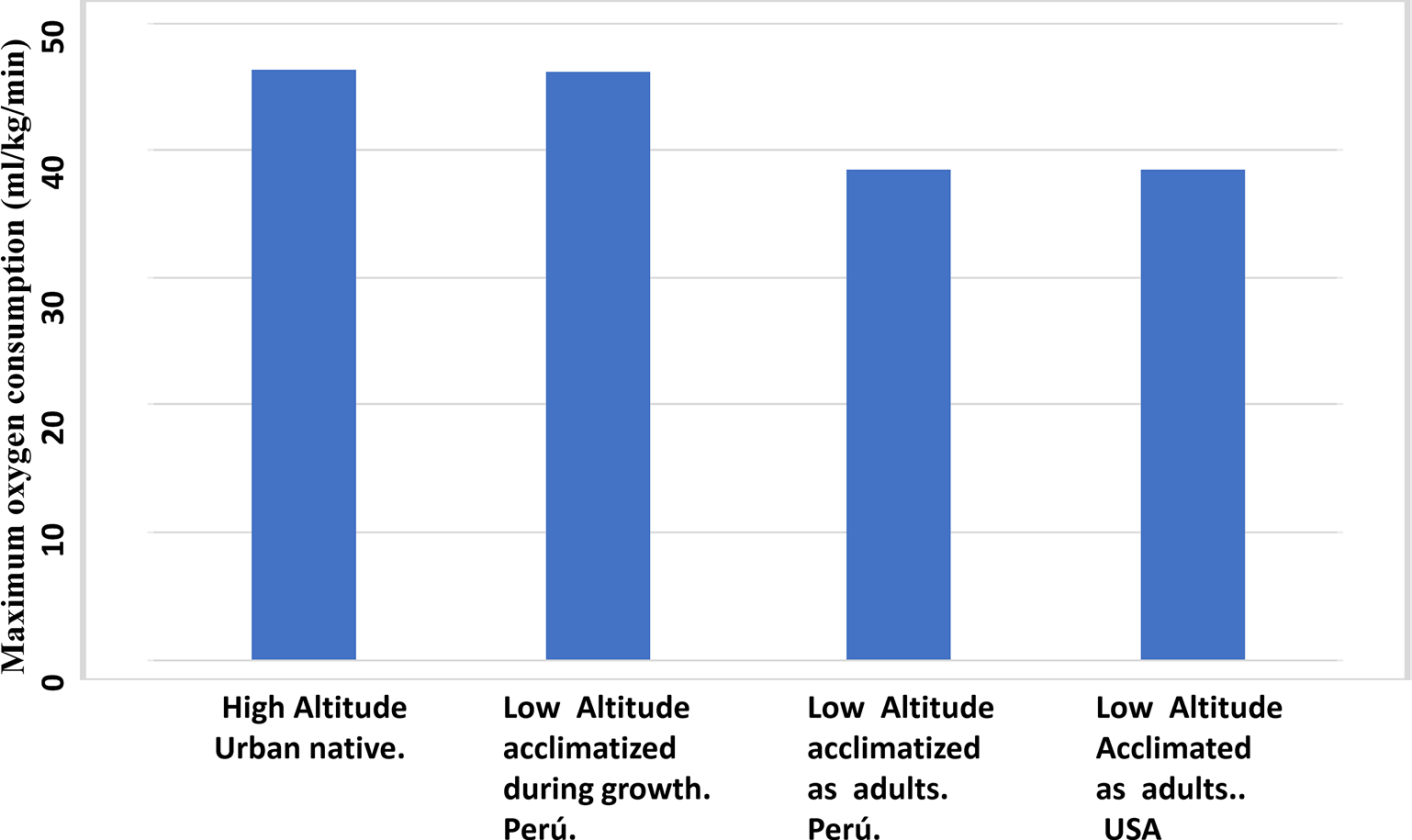
Influence of growth at high altitude on the attainment of high aerobic capacity at an altitude of 3400 m. Participants who acclimatized to high altitude during the developmental period attained similar aerobic capacities as the high‐altitude urban natives. In contrast, the participants from Perú and the United States who acclimatized during adulthood had lower aerobic capacity than the high‐altitude urban natives. *Source:* Adapted from: Frisancho, Martinez et al. (1973); Frisancho (1975).

As shown in Figure 5, the Peruvian low‐altitude natives who, during the developmental period, were acclimatized to chronic high‐altitude attained an aerobic capacity that was equal to that attained by the high‐altitude urban natives. In contrast, both the Peruvian and US low‐altitude natives that migrated to high altitude as adults attained significantly lower aerobic capacity than the high‐altitude urban natives.

#### 4.1.3 Summary: The Role of Developmental Adaptation to High Altitude in Perú

The attainment of the enhanced lung volume and high aerobic capacity of high‐altitude urban natives is influenced by adaptations that occur during the developmental period (see Frisancho, Velasquez et al. 1973; Frisancho, Martinez et al. 1973; Frisancho 1975). However, since most Peruvians are of mixed backgrounds that include Quechua and Spanish ancestry, it is possible that the Quechua ancestry could have contributed to the physiological traits measured in the study done in Cusco. One productive way of ascertaining whether Quechua ancestry contributes to the high functional adaptation of the high‐altitude urban natives is by studying the physiological traits of foreigners who do not share a genetic connection with high‐altitude natives (Mazess 1969). Consequently, to address this issue, I initiated research in collaboration with colleagues at the *Instituto Boliviano de Biología de Altura* (IBBA) in La Paz, Bolivia, studying lung function and aerobic capacity among Europeans who were acclimatized to high altitude during different ages.

### 4.2 Studies of Functional Adaptation to High Altitude in La Paz, Bolivia

The research in La Paz was conducted at the physiological laboratory facilities of the IBBA, situated at a mean altitude of 3752 m. The research participants included male and female Europeans who were acclimatized to high altitude during different developmental ages and during adulthood. Selected physiological variables were measured under resting and exercising conditions.

#### 4.2.1 Evaluation of Lung Volume

The measurements of lung volume included FVC and residual lung volume. The sample consisted of 357 participants and was comprised of: (1) 37 rural high‐altitude natives (all male), (2) 125 urban high‐altitude natives of Bolivia (69 male, 58 female), (3) 85 Bolivians of European ancestry acclimatized to high altitude since birth (40 male, 45 female), (4) 63 Bolivians of European ancestry acclimatized to high altitude during growth (30 male, 33 female), and (5) 47 Bolivians of either European or North American ancestry acclimatized to high altitude during adulthood (24 male, 23 female). Hereafter, all Bolivians of foreign ancestry are referred to as Europeans.

**FIGURE 6:**
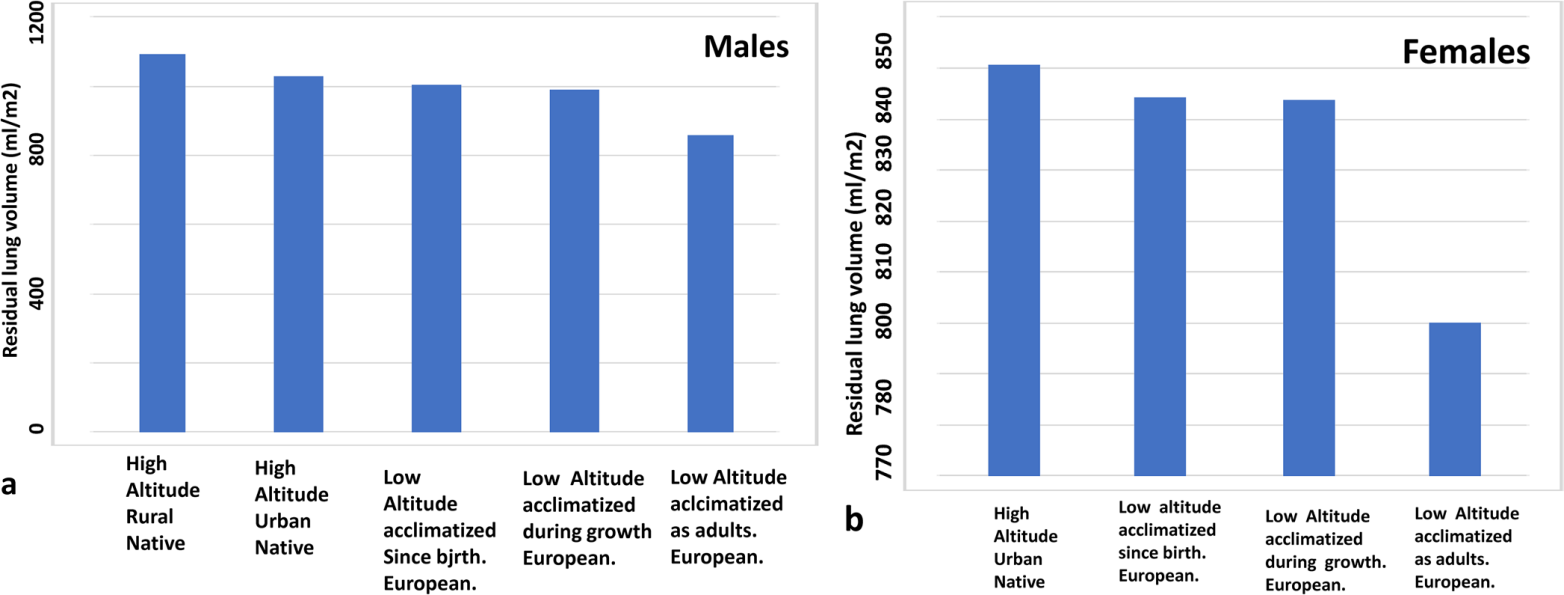
Influence of developing at high altitude on the attainment of high residual lung volume in (a) males and (b) females. European males and females acclimatized to high altitude during the developmental period attained similar residual lung volume as the high‐altitude urban natives from Bolivia, and greater lung volume than those acclimatized during adulthood. *Source:* Adapted from: Frisancho et al. (1997).

The results are summarized in Figure 6, showing the residual lung volumes per unit surface area (mL/m^2^). European males and females acclimatized to high altitude during the developmental period, especially those acclimatized during early growth, attained similar residual lung volumes to the high‐altitude urban natives and had greater lung volumes than those acclimatized to high altitude during adulthood. Additionally, age at arrival to high altitude was inversely related to residual lung volume, suggesting the influence of early adaptation to high altitude.

#### 4.2.2 Evaluation of Aerobic Capacity

The measurements of oxygen consumption under maximal exercise were carried out in the Exercise Laboratory of IBBA. The sample consisted of 268 participants (158 males and 110 females) including: (a) 39 male high‐altitude rural natives, (b) 67 high‐altitude urban natives (32 male, 35 female), (c) 69 Bolivians of European ancestry (37 male, 32 female) acclimatized to high altitude since birth, (d) 50 Bolivians of European ancestry (25 male, 25 female) acclimatized to high altitude during growth, and (e) 43 Bolivians of European or North European American ancestry (25 male, 18 female) acclimatized to high altitude during adulthood.

**FIGURE 7:**
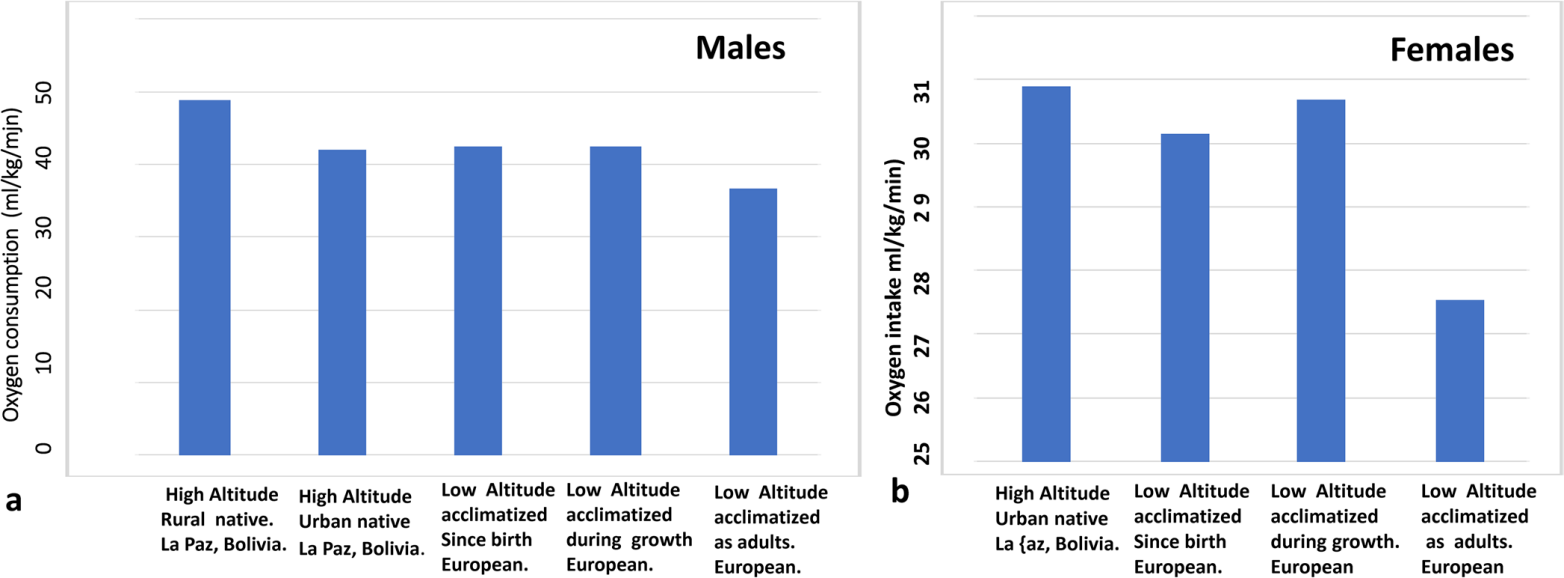
Influence of developmental adaptation on the attainment of high aerobic capacity in (a) males and (b) females. European males and females acclimatized to high altitude during the developmental period attained similar aerobic capacity as the urban natives from Bolivia, and greater aerobic capacity than those acclimatized during adulthood. *Source:* Adapted from: Frisancho et al. (1995).

The results are summarized in Figure 7. Europeans acclimatized to high altitude during the developmental period, especially those acclimatized during early growth, attained a similar aerobic capacity as the high‐altitude urban natives and a higher aerobic capacity than their counterparts acclimatized to high altitude during adulthood. Additionally, the aerobic capacity among participants acclimatized to high altitude before the age of 10 years was greater than that of their counterparts acclimatized later in life. Finally, the aerobic capacity of high‐altitude rural males was significantly greater than that of all of the other groups studied in La Paz.

In sum, the research conducted in Cusco, Perú included Peruvian and US urban natives, whereas the study in La Paz, Bolivia included Bolivian rural and urban natives as well as Europeans acclimatized to high altitude. Together, these studies indicate that functional adaptation to high altitude of urban natives is largely related to physiological characteristics acquired during the developmental period.

Kiyamu et al. (2015) measured arterial oxygen saturation (SaO_2_) during submaximal exercise in Lima, Perú (150 m) under normal and hypobaric conditions among a sample who differed in their developmental exposure to high altitude hypoxia. This study found that those born at high altitudes maintained higher SaO_2_ than their counterparts born at sea level. These findings suggest that the influence of developmental adaptation to high altitude on the efficiency of the cardiovascular system in transferring oxygen from the lungs into the bloodstream is retained even after a long residence at sea level.

## 5 Epigenetic Changes and Developmental Adaptation to High Altitude

Epigenetics refers to the functional modification of the genome. Depending on the environmental stressors and thru the process of methylation the function and expression of the DNA can be modified facilitating or impeding adaptation of the organism. This plasticity is more likely to occur during the developmental period than during adulthood. Recent work has examined the role of epigenetic changes in promoting developmental adaptation to high altitude. Epigenetic methylation is a process whereby small chemical groups (methyl groups) attach to the DNA, modifying its function without altering the sequence. Such changes may facilitate responses and adaptation to environmental conditions.

An international team of research scientists from Germany, the United States, and Perú conducted three major studies to evaluate whether acclimatization to high altitude induces epigenetic changes in the DNA. The first study by Childebayeva et al. (2019a) used quantitative pyrosequencing to analyze DNA methylation of blood samples of 572 Quechua from Perú who were born and raised at high altitude (4388 m), 147 individuals born in high altitude but migrated to low altitude, and 143 born and raised at low altitude. The results indicated that lifetime exposure to high‐altitude hypoxia has an effect on the Endothelial PAS Domain Containing Protein 1 (EPAS1) and LINE‐1 methylation among Andean Quechua, suggesting that epigenetic modifications play a role in high‐altitude adaptation.

The second study by Childebayeva et al. (2019b) evaluated the saliva of 21 participants of European ancestry trekking to high altitude. The study found that there were changes in DNA methylation associated with the ascent to high altitude.

The third study evaluated the genome‐wide epigenetic signatures in blood samples obtained from a total of 113 participants, including: (a) 36 low‐altitude Quechua, (b) 39 low‐altitude Quechua who migrated to high‐altitude, and (c) 38 life‐long, high‐altitude Quechua (Childebayeva et al. 2021). This study identified two differentially methylated positions (DMPs) and 62 associated with high‐altitude developmental and lifelong exposure to high altitude. Furthermore, the investigation found that DMPs were active in genes associated with the hypoxia‐inducible factor (HIF) pathway and the EPAS1 genotypes.

In sum, these studies demonstrate that acclimatization to high altitude, especially during the developmental period, is associated with DNA methylation. Such epigenetic changes facilitate the attainment of full functional adaptation to high altitude.

## 6 Summary and Conclusions

**FIGURE 8:**
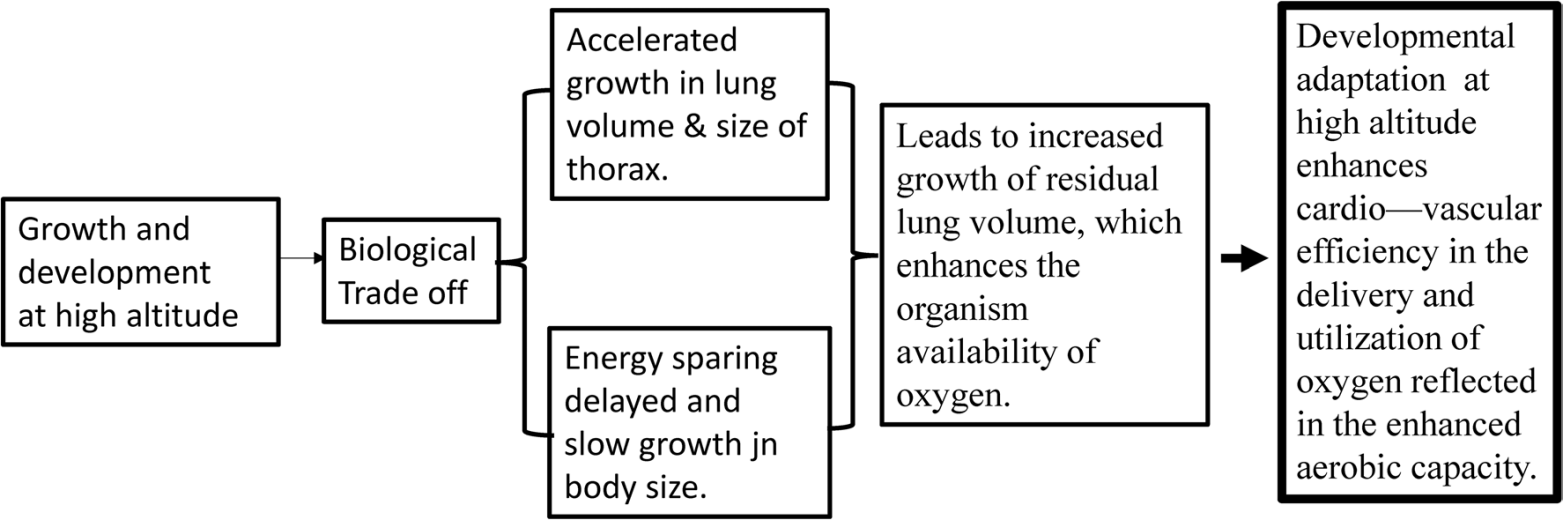
Overview of the role of developmental adaptation to high altitude. Growth at altitude is an important factor that contributes to the attainment of full functional adaptation to high altitude.

Based on the study of human growth in body size and lung volume in Nuñoa‐Perú and the physiological studies of urban samples from Cusco‐Perú and La Paz,‐Bolivia, we developed the conceptual framework of developmental adaptation that maintains that the large lung volume and high aerobic capacity of high‐altitude urban natives are largely the result of adaptive responses that occur during the period of growth and development. As summarized in Figure 8 growth at altitude induces a biological trade‐off characterized by an accelerated lung growth and slow growth in body size. The accelerated lung growth promotes enlarged residual lung volume.

The physiological studies subsequently carried out in Cusco, Puno of Perú, and La Paz of Bolivia advanced our understanding of these adaptive processes, documenting that acclimatization during development among *both* Andeans and Europeans leads to the attainment of enlarged lung volumes and enhanced aerobic capacity. This evidence indicates that the functional adaptation to high altitude is shaped by physiological adaptive processes that occur during the developmental period.

Epigenetic studies demonstrate that acclimatization to high altitude, especially during the developmental period, is associated with DNA methylation. These epigenetic changes facilitate the attainment of similar functional adaptation as observed among high‐altitude urban natives. The conceptual framework of developmental adaptation originally advanced in high‐altitude research is now widely used by health researchers examining “Developmental Origins of Health and Disease” (DOHaD) (e.g., Kuzawa 2007; Kuzawa and Sweet 2009). This domain of research is applying the insights and principles from developmental adaptation to explore the influence of environmental factors during prenatal and postnatal development on physiological and metabolic health outcomes during adulthood (see Frisancho 2009). The concept of developmental adaptation is currently referred to as developmental plasticity and is widely used by health researchers to account for the influence of environmental factors during prenatal and postnatal development on physiological and metabolic differences during adulthood.

### 6.1 Future Research

Current research demonstrates that functional adaptation among high‐altitude urban natives is largely the result of processes during the developmental period. However, it is not known how genetic factors interact with the physiological adaptation that occurrs during development of high altitude rural natives. Hence, future genomic studies that identifies genes that are involved in oxygen homeostasis, cardiovascular function, red blood cell production, and oxidative stress (Caro‐Consuegra et al. 2022; Bigham et al. 2013; Valverde et al. 2015; Moore 2017; Beall 2007; Simonson et al. 2015) are vital for ascertaining their link to reproductive traits and functional adaptation of high altitude Andean rural natives.
